# The Architecture of Public Buildings as a Transformative Model Toward Health and Sustainability

**DOI:** 10.3390/ijerph22050736

**Published:** 2025-05-07

**Authors:** Mihajlo Zinoski, Iva Petrunova, Jana Brsakoska

**Affiliations:** 1 Faculty of Architecture, “Ss. Cyril and Methodius” University in Skopje, 1000 Skopje, North Macedonia; brsakoska.jana@arh.ukim.edu.mk; 2 Association for City Research—GRADOT UBAV Skopje, 1000 Skopje, North Macedonia; iva_petrunova@yahoo.com

**Keywords:** public buildings, sustainability, transformation, healthcare facilities, public–private partnership

## Abstract

Public buildings are crucial to creating healthy and sustainable cities. These buildings promote social cohesion and enrich urban life by transforming existing facilities into hybrid models that integrate medical content. Historical developments highlight shifts in residential, economic, and healthcare infrastructure. The healthcare system aims to enhance public health while ensuring financial equity. Reforms in healthcare privatization, governed by public health and insurance policies, involve liberalizing service provision and are supported by the Ministry of Health and Finance. This study examines how public buildings can adapt to enhance health and social sustainability. Through case studies, it assesses architectural adaptability in analyzing spatial, economic, and social impacts. Diagrams illustrate spatial dynamics, while surveys compare efficiency, sustainability, and user experience. Statistical analysis highlights the role of spatial adaptability in fostering sustainable urban environments. The results, which express significant differences between means for different locations and citizens’ satisfaction, suggest that the hypothesis offers substantial results in every area. Besides commercial programs in commercial buildings, healthcare also gives satisfactory results. This study advocates for adaptive architecture as a key strategy, aligning with evolving societal and health demands. Hybridizing healthcare facilities and commercial spaces transforms shopping centers into sustainable models, enhancing social cohesion and economic viability.

## 1. Introduction

A healthy city is more than a place where people live; it is an ecosystem that supports its inhabitants’ health, well-being, and sustainability. Public buildings play a key role in this vision through innovative architectural approaches that create spaces for social interaction [1]. Social sustainability in urban environments implies a holistic approach to urban planning, where public buildings are integrated with the transport system, green spaces, and municipal services. For example, schools and health centers can be designed to support local communities through energy efficiency, accessibility, and multifunctionality [2].

The functionality and symbolism of public buildings address contemporary challenges such as climate change and citizens’ basic needs and serve as drivers and catalysts in transforming processes in cities’ urban environments into just, healthy, and sustainable urban environments. Analyzing the role of public buildings in creating healthy and sustainable cities through the case of Skopje, this paper will explore how commercial public buildings can contribute to climate resilience, public health systems, social equity, and economic sustainability. By using an innovative approach of Multi-Sector Partnerships in contemporary pluralistic societies, this study acknowledges that commercial buildings can be a transformative model that establishes a sustainable and urban environment for citizens.

### 1.1. Public Buildings as a Social System

A public health facility is oriented around environmental diseases and health challenges. Communication about these diseases becomes part of the health system, and buildings play a key role. Public buildings are “subsystems” within the broader social system. They provide space for social interactions, access to services, and promote equality. A more systematic approach is needed in public architecture to establish a stronger connection between theory, research, and practice. This implies that sustainability and health aspects in architecture should be built based on verified evidence, not just on information or subjective evaluations, to significantly impact users’ health and well-being [3].

Organizations such as hospitals must preserve their essential social identity in complex environments. If a hospital loses its character as a health institution, transforming into a factory, school, or commercial entity, it can no longer be part of the health system. This means that it cannot create recognizable and legitimate communications in the context of health codes and programs. Poly-contexturality carries the potential for conflict and tension, but it is also necessary for dealing with the complexities of the environment. Hospitals, by selecting relevant issues and ways of dealing with them, reduce complexity both in their environment and internally. This allows them to remain functional and respond appropriately to different needs [4].

The connection between organizations and functional systems is significant because organizations are the only social systems that can communicate with their environment. In doing so, they preserve operational independence but enable connectivity and coordination with other organizations. Some public facilities may be managed through Public–Private Partnerships (PPPs), where private companies develop, maintain, or modernize facilities in collaboration with state institutions [5].

The design should integrate different contexts (health, education, and commercial) in public building architecture without compromising the primary purpose. In this way, public buildings can contribute to sustainable and healthy solutions while simultaneously adapting to the complex needs of modern society [4].

### 1.2. Transformation of Societal Institutions

Contemporary architecture is critical in responding to urbanization, climate change, and social injustice. Public buildings, especially those in the healthcare sector, need to be designed as integrative platforms that enable positive impacts on human health, ecosystems, and social cohesion. The architecture of public buildings can employ transformative strategies to address contemporary communities’ needs. This study hypothesizes that new hybrid public structures can create healthy and sustainable conditions for people and nature.

Promoting commodities and public commercial buildings can be designed to encourage inclusive healthcare services. In recent years, Public–Private Partnerships (PPPs) have been constructively considered for reforming health sectors in many countries [5].

The potential of public buildings for transformation is seen in social inclusiveness (public buildings such as hospitals, schools, and libraries should be places where all social groups can feel welcome) and in the democratization of space (public buildings should promote social interaction, avoiding isolated “ghettoized” communities).

Access to healthcare requires various delivery methods and services that meet the needs of communities. An increasing number of healthcare services are being delivered outside traditional hospitals and clinics to facilities such as community centers, wellness centers, and even patients’ homes. Healthcare services in nearby shopping centers are closer to communities, better serve their needs, and are more cost-effective to build and operate. These alternative locations improve access to healthcare and contribute to sustainability by reducing the carbon footprint of transportation, construction, and operations. The flexible design and integration of disparate commercial functions demonstrate shopping centers’ poly-contextuality. Private hospitals and clinics that combine healthcare services with opportunities for exercise, socialization, and organic markets can contribute to greater accessibility, cost-effectiveness, and sustainability while meeting communities’ diverse needs [6].

Liberalization of healthcare services has often redefined how public buildings are created and used. With its orientation toward free markets, privatization, and the minimization of the state’s role, it has made medical services that favor the upper socioeconomic classes and exclude marginalized groups accessible, creating the potential of architecture to be a catalyst for social transformation.

This study is organized into three chapters, each addressing the healthcare decentralization and establishment of Public–Private Partnerships (PPPs): the spatial transformations of public buildings, the integration of healthcare facilities into commercial centers, and a qualitative exploration of the aspects contributing to social sustainability. The first chapter provides a literature review of theories. It establishes a methodology for study scope, including studies that discuss the sustainability of PPPs in healthcare and accessibility of the newly distributed network of healthcare facilities. Moreover, it elaborates on the Macedonian healthcare system and the redistribution of healthcare services in other facilities. The second chapter explores the methodology, detailing the approaches and methods utilized in this research. It presents an overview of the case studies of three shopping centers in Skopje.

The third chapter showcases the results, summarizing the case studies’ data and data on analyzed social outcomes and user satisfaction regarding distributed healthcare facilities through a survey. The concluding chapters include the conclusion and discussion and present the final observations.

## 2. Literature Review

### 2.1. Theoretical Framework

Urbanization and political transformations during the 19th and 20th centuries created a need for public spaces and buildings to foster social integration and address structural challenges. In the context of German modernization, these spaces were instrumentalized to overcome class differences and create a sense of community. Although often contested, this approach significantly contributed to cities’ political and cultural development [6].

Sociologist Ferdinand Tönnies analyzes the relationship between “community” and “society” in urban environments, emphasizing that public buildings can be a platform for overcoming the mechanical relationships that characterize modern cities. This positions architecture as a mediator between individual and collective interests while at the same time encouraging sustainable and healthy ways of living [7].

Public buildings can be viewed as systems that distinguish themselves from their environment as part of a broader social system. According to Luhmann [4,8], a system exists only by differentiating itself from its environment. In the context of public buildings, their function is oriented toward providing services that respond to the needs of their environment (Table 1).

According to Luhmann, systems create an internal representation of the environment that affects them. This means that public buildings can be designed to reflect the community’s needs, such as physical health, mental well-being, and sustainability [4].

According to Luhmann’s theory of autopoiesis, healthcare functions as a self-sustaining system that creates and reproduces its communications and values through legitimate sources. This indicates that public buildings should also be based on clearly defined principles and communication, which will enable the reproduction of healthy and sustainable practices. By creating systemic, validated solutions, architecture can become an independent mechanism that constantly supports and improves health and environmental standards [4,8].

According to Botchwey et al. [6], hospitals like Kaiser Permanente are creating new typologies, combining healthcare services with exercise spaces, markets, and cultural activities, which reduces the gap between the healthcare system and the community [6].

Key sustainability practices include location and accessibility (facilities close to communities to minimize transportation and increase accessibility) and multifunctional healthcare spaces (smaller clinics/health premises in local communities or shopping malls that reduce the need for travel and create local health centers). According to Botchwey [6], hospitals can be catalysts for sustainability by functioning effectively and promoting a healthy lifestyle in the community.

### 2.2. Main Approach of the Scoping Review

**Figure 1:**
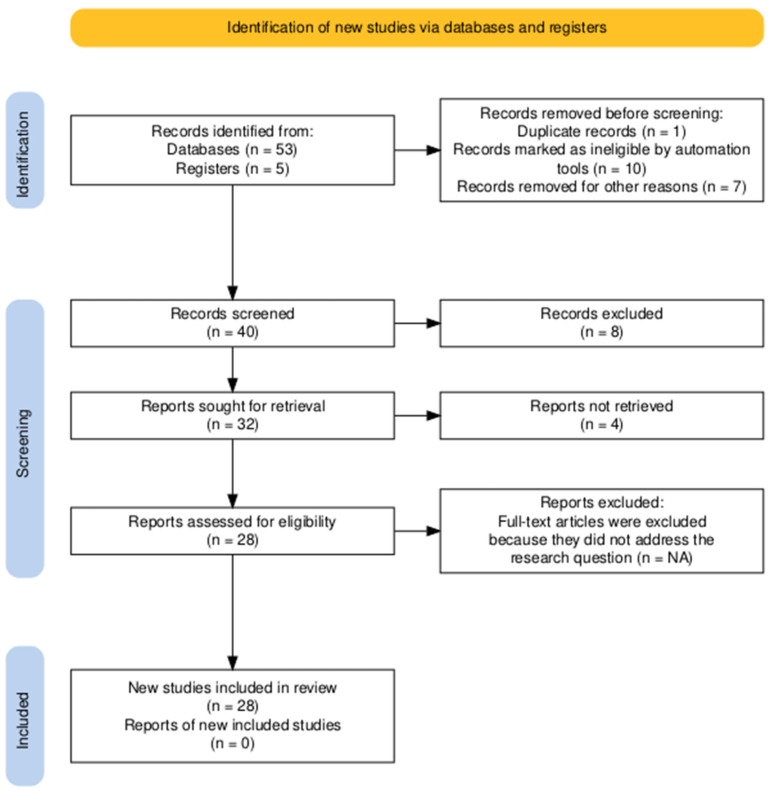
Scoping review methodology flowchart by PRISMA-ScR flowchart. Source: https://doi.org/10.1002/cl2.1230.

This scoping review is structured according to the PRISMA framework. It follows the PRISMA-ScR guidelines, an extension of the Preferred Reporting Items for Systematic Reviews and Meta-Analyses (PRISMA) method designed explicitly for scoping reviews. After establishing the inclusion and exclusion criteria, the PRISMA flow diagram illustrates the scoping review (Figure 1). This stage involved a comprehensive assessment of relevance, reviewing, and selecting appropriate studies. The figure highlights the structured approach employed to systematically identify and evaluate the literature, ensuring a rigorous and thorough review process [9].

Despite extensive studies on topics such as social sustainability in healthcare, Public–Private Partnerships (PPPs) in healthcare, health facilities in commercial buildings, accessibility, and community health, a significant gap remains in our understanding of the specific transformations of public buildings and the integration of healthcare facilities within commercial buildings following PPP reforms. The existing literature highlights the broad impact of PPPs and accessibility on healthcare systems, overall well-being, and social sustainability in healthcare [5,10,11,12,13,14,15,16,17,18,19,20,21,22,23,24,25,26,27,28,29,30,31,32,33,34,35].

Public healthcare buildings need to be reimagined as inclusive spaces. Planning multifunctional buildings that combine health, education, and social functions can revitalize public space. To benefit from the capabilities of the public and private sectors in the form of a hybrid model, the Public–Private Partnership (PPP) model was introduced in the 1990s, and it has been used constructively in recent years to reform healthcare sectors in many countries. The PPP model can be a powerful political tool for improving and promoting the survival and quality of services in public hospitals [5,30]. The studies of PPP highlighted the importance of effective implementation strategies and equity considerations in enhancing population health outcomes through such partnerships [30,31,32,33,34,35,36].

Achieving social sustainability in healthcare facilities through the adaptive reuse of public buildings presents a challenge that combines architectural innovation with community well-being. Previous studies have evaluated social sustainability in healthcare by focusing on humanization, comfort, patient demand, satisfaction, financial success, and accessibility criteria [18,19,20]. Several reports [29] highlight the built environment’s impact on health and well-being, indicating that the condition of the built environment can create opportunities for social activities, social infrastructure, and access to facilities that influence health across the lifespan [29].

To understand how accessibility (analyzed in this study in terms of proximity related to time and distance) to private health centers located in commercial buildings affects public health, we can draw insights from various studies and observations. Weinberger et al. [10] assert that accessibility and convenience are significantly improved when private health centers are situated in commercial buildings. Locations like malls and supermarkets are often more accessible than traditional hospital campuses and medical office buildings, making it easier for individuals to seek care. This improved accessibility leads to more convenient healthcare options and higher patient satisfaction. As healthcare becomes more focused on consumers, clinics situated in retail environments offer opportunities for expansion and greater community involvement. Clinics in commercial settlements have become more attractive because mall centers generate additional foot traffic [10].

Studies on accessibility have demonstrated that better access to healthcare facilities positively impacts health outcome indicators, where the proximity of these facilities is crucial for enhancing health outcomes [21,22,23,24,25,26,27]. However, the distribution of these facilities must be equitable. Ensuring that healthcare facilities outside institutional medical centers are evenly distributed and accessible to all population segments is vital for maximizing their impact on public health [10,11,15].

Given the pressing need to improve quality while reducing costs, healthcare policymakers and funding authorities are increasingly focused on optimizing the allocation of healthcare facilities by attempting to (re)distribute them more effectively [16].

### 2.3. The Healthcare in North Macedonia

In the early 21st century in Europe and the US, decentralization began to mean abandoning the building of larger and more consolidated healthcare facilities and replacing them with networks of mostly smaller facilities [37]. These processes have raised the question of which model provides better patient-centered healthcare, where the answer may be the combination of both models—centralized and decentralized. Moreover, it refers to the concentration of specialized care in large hospital complexes, the decentralization of medical consultation, and lower-level medical interventions as single-doctor operations [38]. The healthcare system has its particular way of distributing medical services. The reforms of privatization and de-privatization of healthcare financing methods are processes aimed at decentralization, achieving a balance between healthcare costs and actual financial capabilities, and improving community health. The reforms in Macedonia were mainly initiated and guided by the World Bank’s Health Sector Transition Project and the Health Sector Management Project. The healthcare system in Macedonia has developed a social health insurance system. Retaining the positive features of the previous Yugoslav health system, the healthcare system became highly centralized at that time. After several reforms, healthcare service provision was liberalized, enabling private providers to enter it [38]. In 2007, only three years after the reforms, 95% of the licensed primary healthcare physicians had moved to the private sector [39]. Private investment in secondary and tertiary healthcare levels occurred before government reforms intended for these areas. As a result, private general and specialized hospitals began to separate themselves from centralized public hospital care, providing services outside the health insurance system that users are paying for directly. Moreover, the primary care practices established a Public–Private Partnership (PPP) with the Health Insurance Fund (HIF), where a capitation-based payment replaced the previous salary-based payment system for doctors, with the income linked to the number of citizens enrolled on primary care providers’ lists. Capitation-based payment is based on a blended-capitation model, which includes a 70% fixed amount and a 30% variable amount. This means fulfilling obligatory preventive health activities, rational prescribing and referral, preventive check-ups, and health promotion and education activities [40]. In the transition period after the SFRY collapse, Skopje, the capital city of Macedonia, faces transformations toward a pluralistic and liberal political economy. In transition societies today, it is evident that the economy acts politically, but its politics ultimately aims to establish economic criteria as the primary organization of the human environment (Table 2). After the Industrial Revolution, the global economy transformed into a world of economy [41].

Becoming weak, the national economy finds the solution to be selling state property to private investors domestically and abroad. Public real estate becomes the subject of privatization. Influenced by insufficient state funding, primary and secondary national healthcare systems started to shift toward private healthcare [42,43]. Moreover, the Macedonian healthcare system and the Ministry of Health determine the Health Insurance Fund (HIF), with a purchaser–provider split and a mix of public and private providers. After several reforms, the healthcare service provision market was liberalized, enabling private providers to enter the market [38].

Building brand-new hospitals and clinics is a demanding process regarding their location properties, price, working and building permissions, qualified medical personnel, expensive equipment, and environmental congestion they create. In contrast, adapting existing facilities proves to be a more economically sound approach, as healthcare practices typically have standard requirements for space and specific areas of operation [44]. It is necessary to emphasize the Multi-Sector Partnerships. It makes the existing cooperation more diverse and realistic, thus considering the social aspect, which brings it closer to the complexity of real urban ecosystems.

In the participation process, the public and private are usually facilitators and providers, and the participation of the people is as end-users, respectively. Participating entities provide assets or services according to their characteristics [5,30].

Entering healthcare services alongside the existing tenant mix of commercial commodities into shopping centers, this phenomenon of poly-contextuality turns shopping centers into local community centers. This adaptation opens up new possibilities for healthcare policymakers and creates opportunities for private practices to contribute to the employment of medical professionals and staff.

Hybridization becomes a transformative model with recognizable social and economic benefits for the parties involved. Existing local community centers are near the neighborhoods, easily accessible to the citizens, and contribute to the social sustainability of the existing communities in Skopje.

## 3. Materials and Methods

This article presents exploratory qualitative research and descriptive analysis to increase our understanding of the hybridization phenomenon in public shopping centers. Since the transformation of shopping centers from commercial to healthcare services has not been defined as a problem in exploratory research by analyzing theoretical data during the literature review stage, the researchers use data about the PPP model and its development in polyclinic services in Skopje from research undertaken previously; the literature scoping review benefits from the PRISMA framework structure.

The researchers gather new data for descriptive research through case studies, surveys, and observations. Descriptive research through the direct observation method incorporates two sources of data: analyzing the spatial characteristics and working conditions in the facilities of the case studies through a questionnaire and a survey of the space users. User satisfaction is assessed through a study and analyzed using the statistical technique Analysis of Variance (ANOVA).

### 3.1. Literature Review on Methodology

Previous studies have focused on evaluating social sustainability and PPP models in healthcare, notably through how the built environment affects health and well-being. Standard methods of the literature scope include analytical approaches, such as interviews with focus groups from various fields [18,19,20]. ANOVA is often used as a statistical technique, along with empirical quantitative methods involving questionnaires [14]. Additionally, other studies have employed proposed indices to investigate the spatial accessibility of public health service facilities within the study area [15]. Furthermore, accessibility is often illustrated through diagrammatic representations or techniques that visualize and analyze spatial data for mapping the case study [17].

### 3.2. Exploratory Qualitative Research Analyses

The theoretical framework is the foundation for gradually revising existing architectural theories and frameworks that address social systems and sustainability. The PRISMA ScR method is implemented in five stages in the scoping review. The first stage focuses on identifying records from various databases and registers using the following keywords: “social sustainability in healthcare”, “Public–Private Partnerships in healthcare”, “health facilities in commercial buildings”, “accessibility”, and “community health”. Next, relevant studies are identified through a screening process, where the retrieved reports are evaluated for their eligibility. The final stage involves summarizing and reporting the studies included in this review.

Additionally, exploratory analyses are conducted to describe the healthcare system in Macedonia and the reforms that led to establishing Public–Private Partnership (PPP) models in healthcare.

### 3.3. Descriptive Research Through Case Study, Survey, and Observation

#### 3.3.1. Case Studies and Diagram Illustration: The Transformation of Open Shopping Centers in Skopje, Macedonia, into Medical Facilities

The Health Network, established in 2012, consists of public and private healthcare providers. Moreover, since 2013, it has been regulated by e-services via the platform “My Appointment” [45,46]. According to this platform, in the three biggest municipalities in Skopje—Aerodrom, Karposh, and Center—there are approximately 209 private practices in general healthcare and dentistry. A total of 9% of those practices are still accommodated for in public facilities such as Public Health Centers. In total, 91% are distributed in residential and other commercial spaces, of which 4% are located in public buildings such as shopping centers (Table 3).

**Figure 2:**
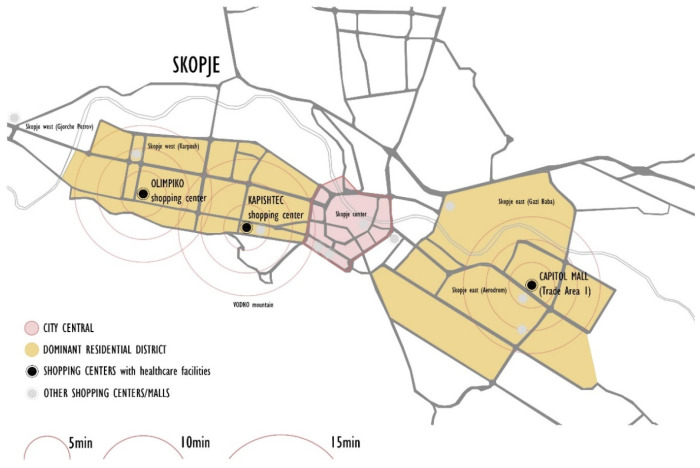
Proximity of healthcare facilities related to walking distance. Source: authors, 2025.

**Figure 3:**
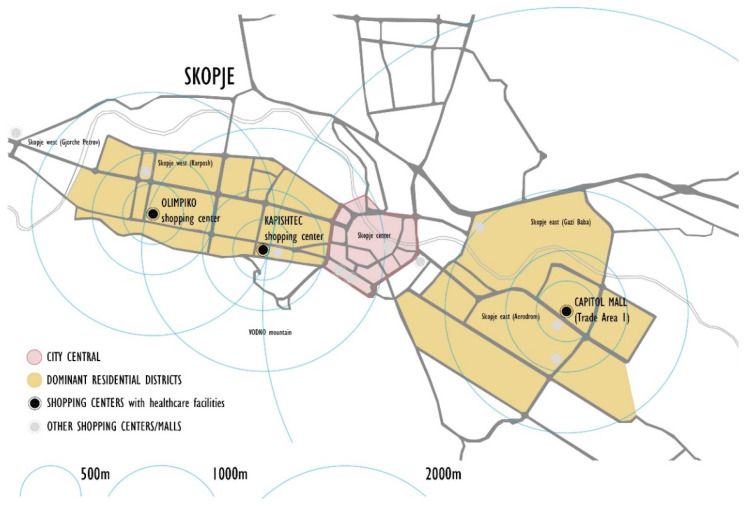
Proximity of healthcare facilities related to walking time. Source: authors, 2025.

Today, at least 4% of private healthcare facilities are accommodated in shopping centers in three municipalities in Skopje. Medical amenities such as disparate programs in shopping centers exemplify the phenomenon of poly-contexturality. This co-existence has advantages regarding proximity within walking distance in dense neighborhoods (Figure 2). This proximity is convenient in shortening the time to reach the medical service (Figure 3).

The descriptive research method focuses on the Public–Private Partnership (PPP) models of dispersed healthcare practices in shopping centers. This research is conducted through a questionnaire directed at medical professionals and staff who manage and work at these practices. These groups of respondents are the target audience for this study, and their responses are presented in tabular form.

The survey questionnaire is divided into eight sections, and data are collected from three shopping malls, specifically at eight outpatient clinics. The questions address the structure of the practices, the number of employees, the available medical care and services, and the factors influencing the decision to choose their location. The responses are grouped in categories according to the type of medical practice. Data are collected from medical practitioners from primary and secondary healthcare practices, regardless of their ownership status.

#### 3.3.2. Survey (One-Way ANOVA)

The survey (one-way ANOVA) can be applied in the following ways:

- Assessing Spatial Efficiency of Transformations: Different case studies (e.g., various shopping centers converted into medical facilities) can be compared based on spatial efficiency. Metrics such as patient capacity, space utilization rate, and accessibility improvements can be analyzed to determine whether certain transformations are significantly more effective.
- Evaluating Environmental and Sustainability Impacts.
- Analyzing Social and Functional Outcomes: User experience surveys, foot traffic analysis, and public satisfaction data.

In the context of this study, the ANOVA technique uses a survey method to investigate citizens’ satisfaction with the presence of medical amenities in commercial buildings.

The survey consists of three groups of questions:

- The first set of eight questions evaluates the presence of existing healthcare facilities near the respondent’s place of residence.
- The second set of four questions assesses the respondent’s desire for healthcare facilities to be located within existing shopping centers near their residence.
- The third set of five questions examines the actual presence of healthcare facilities in existing shopping centers.

Citizens’ answers from the survey were collected using a Likert scale. Respondents rated their level of agreement or disagreement with the question on a symmetric agree-disagree scale.

The target respondents were passersby at selected shopping centers who lived in the immediate vicinity. Out of 65 answered questionnaires, in terms of demographic data, 57% are aged 20–45, while 43% are aged 45–75. Furthermore, 74% of respondents live near shopping centers, the remaining 24.5% are at a distance of approximately 3.5 km, and only 1.5% are at a distance greater than 5 km. In total, 90% of respondents are users of shopping centers, while the remaining percentage are tenants in residential areas in the immediate vicinity or within the shopping centers.

The data collection is at six characteristic locations, namely the following:

Shopping Center Olimpiko; City Mall Skopje; Shopping Center Kapishtec; City Shopping Center—GTC; Trade area 1—Capitol Mall; East Gate Mall Skopje.

Three of these shopping centers are analyzed in detail through diagrammatic case studies, illustrating the percentage of integrated healthcare facilities. The remaining three centers were found to lack integrated healthcare facilities.

Surveys were conducted at various times, primarily between 10:00 a.m. and 2:00 p.m. and 4:00 p.m. and 6:00 p.m.

Through the ANOVA, this study employs a qualitative research approach using a structured survey to collect data from users, such as visitors and neighborhood residents. The questions evaluate citizens’ overall satisfaction with public building environments and commercial spaces in shopping centers transformed into healthcare amenities. This approach allows for comparing multiple groups based on key variables and variations in design strategies.

## 4. Results

This chapter presents the results of the two methodological approaches used in this study. The first subsection discusses three case studies illustrated through diagrams. The shopping centers, part of Skopje’s built environment, serve various functions, including healthcare services. The diagrams depict the content of the shopping centers, their flexibility in accommodating healthcare programs through adaptive spaces, and the interactions among healthcare users. These diagrams are based on data collected from the previously distributed questionnaire.

The second subsection provides the results of the ANOVA survey, which employed statistical techniques through a questionnaire to evaluate assumptions regarding the social benefits of healthcare services in the shopping centers.

### 4.1. Results from the Case Studies in Skopje

The case study focuses on three shopping centers in Skopje, North Macedonia, specifically within the city’s three largest municipalities: Karposh, Aerodrom, and Center. The first shopping center discussed is Olimpiko, situated in Karposh. The second is Kapishtec, located in the city center. Finally, the third is Shopping Area 1, a mixed-use residential and commercial block development on the north side of Capitol Mall in the Aerodrom municipality.

#### 4.1.1. Results from the Survey

For a detailed analysis of the case studies, a questionnaire was administered to the management and employees of healthcare facilities in shopping centers. The responses (Table 4) qualitatively compared the decision factors and challenges of running small primary healthcare medical practices versus larger secondary healthcare polyclinic practices. Key factors influencing the sustainability of primary care practices include favorable rental prices or ownership options and proximity to the neighborhoods they serve. This is particularly important because small primary care practices collaborate with the Health Insurance Fund (HIF) through Public–Private Partnerships (PPPs), which helps secure their financial stability by increasing patient attendance and numbers. In contrast, larger practices that also provide secondary healthcare depend more on the location and area of their premises within the shopping center and the benefits of the existing communal infrastructure, which facilitates their operations. Furthermore, most practices prefer not to offer walk-in immediate care services. Instead, they primarily operate through private appointments or the public appointment channel called “My Appointment”.

The results emphasize the advantages of location in shopping centers when healthcare facility management is making selections. A prime location significantly contributes to practice development and growth, enhances patient care, and supports the employment of medical staff.

#### 4.1.2. Diagram Illustrations of Three Case Studies in Skopje

- The shopping center “Olimpiko” is in the Karposh municipality, Skopje.

**Figure 4:**
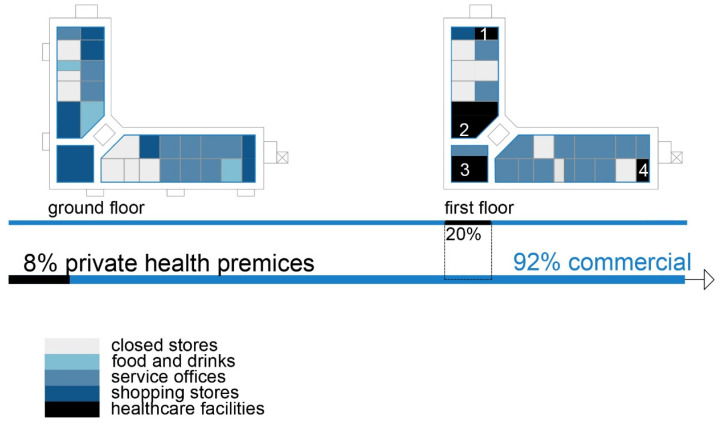
Diagram of SC Olimpiko: Illustration of the building’s spatial content and functional characteristics. 1–4 Health care services. Source: authors, 2025.

**Figure 5:**
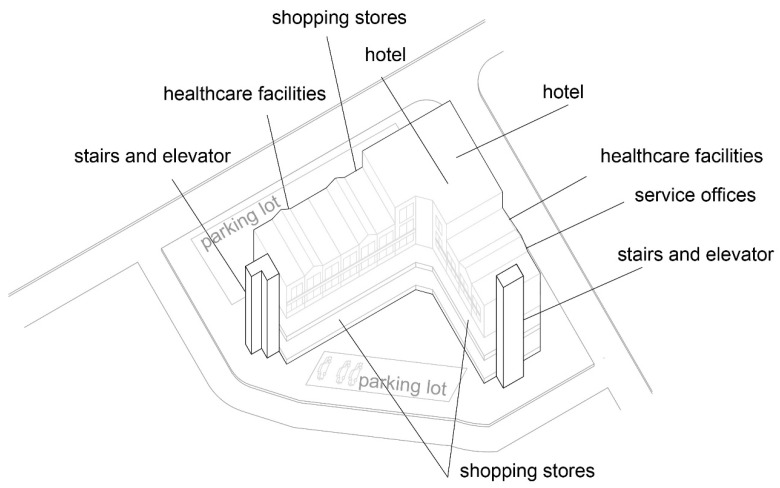
Isometric diagram of disparate content in the SC Olimpiko. Source: authors, 2025.

**Figure 6:**
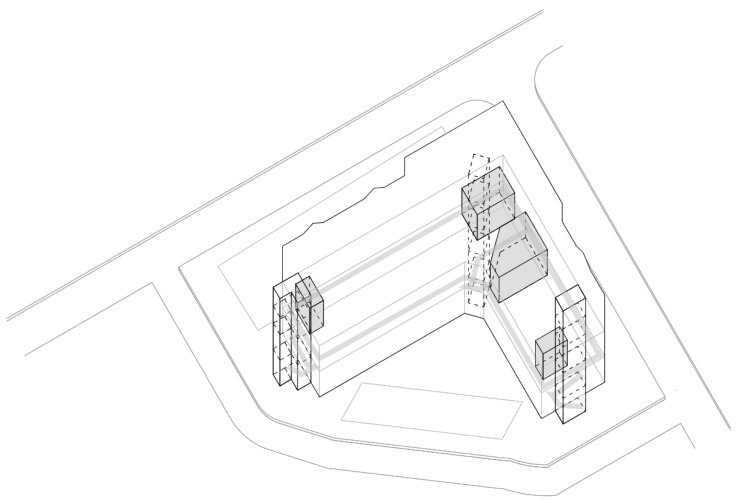
Isometric diagram of circulation in the SC Olimpiko. Source: authors, 2025.

Shopping center: Olimpiko;

Location: Taftalidze 2 neighborhood, municipality of Karposh;

Year built: 1994;

Healthcare facilities: Primary healthcare, specifically general healthcare and dentistry (Figure 4 and Figure 5) (Table 5);

Social interaction: The shopping center has an organized infrastructure that enables a vehicle and pedestrian approach, with sufficient parking lots in its front and back yards. Public transport is available within a 3 min walk. The inner circulation of the shopping center allows patients to directly approach the healthcare premises without a more prominent intersection with the rest of the visitors’ movements (Figure 6).

- The shopping center “Kapishtec” case in the Center municipality, Skopje.

Shopping center: Kapishtec;

Locations: Kapishtec neighborhood, Center municipality;

Year built: 1987

**Figure 7:**
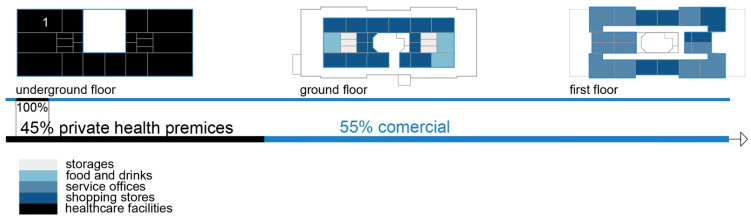
Diagram of SC Kapishtec: Illustration of the building’s spatial content and functional characteristics. 1—Health care services. Source: authors, 2025.

**Figure 8:**
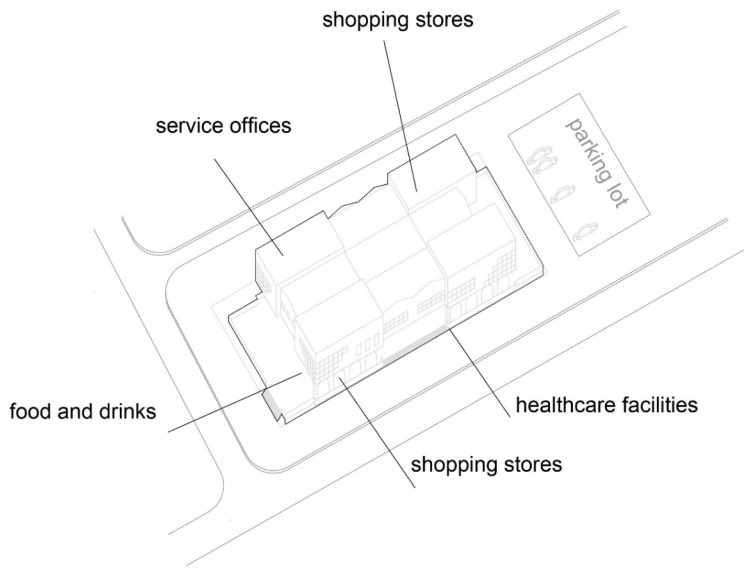
Isometric diagram of disparate content in the SC Kapishtec: Source: authors, 2025.

**Figure 9:**
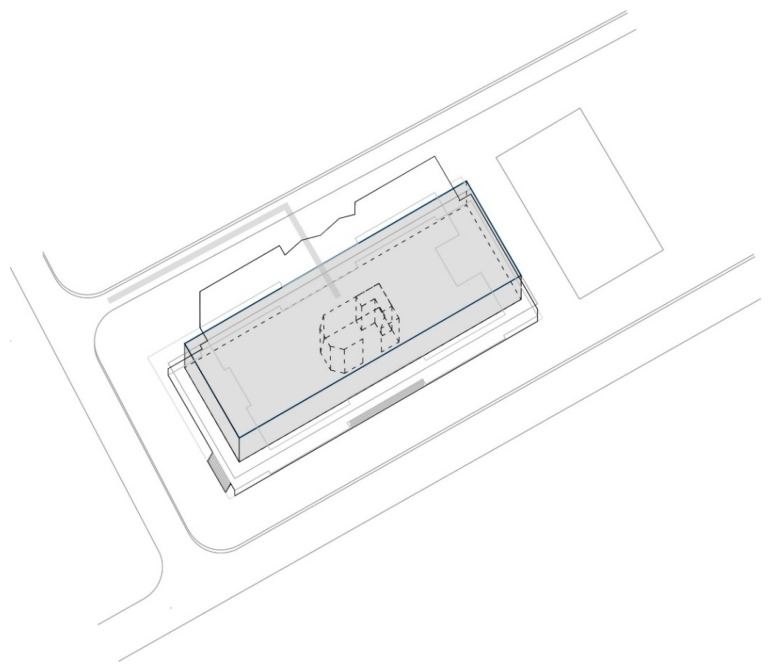
Isometric diagram of circulation in the SC Kapishtec: Source: authors, 2025.

Healthcare facilities: The shopping center has recently accommodated private health facilities for primary and secondary healthcare. A polyclinic occupies the underground level (Figure 7 and Figure 8) (Table 6);

Social interaction: The shopping center has a well-established circulation of vehicles and pedestrian movement. A parking lot and an additional service street enable an approach to the underground level. Public transport is only two minutes away on foot. The polyclinic at the subterranean level has a direct approach for patients and medical staff without compromising the movement of other visitors (Figure 9).

- The “Trade Area 1” case is next to Capitol Mall in Aerodrom municipality, Skopje.

**Figure 10:**
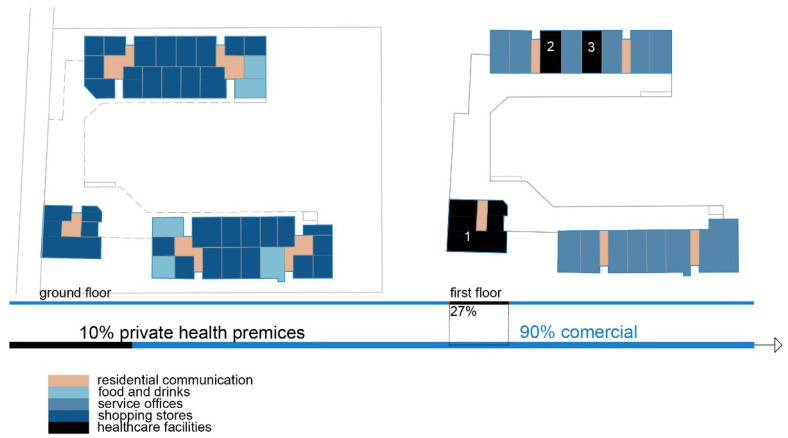
Diagram of TA 1-Capitol: illustration of the building’s spatial content and functional characteristics. 1–3 Health care services. Source: authors, 2025.

**Figure 11:**
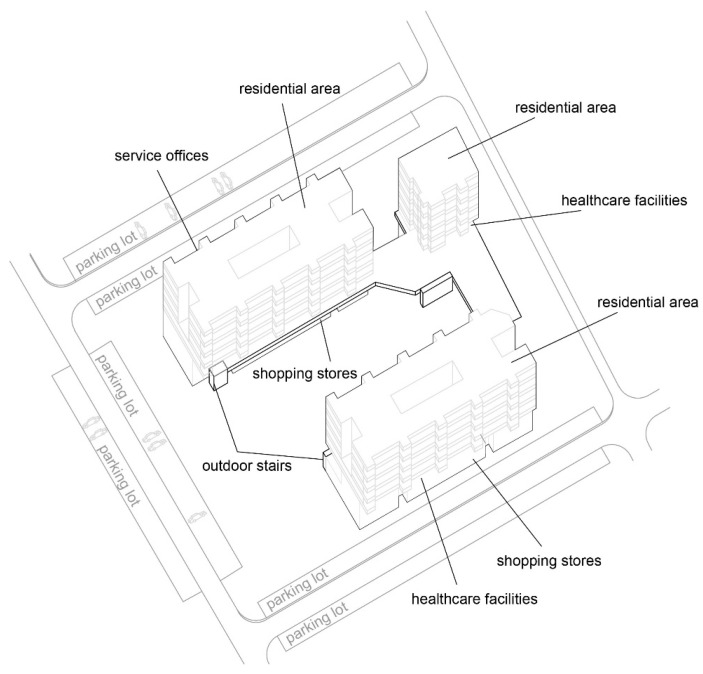
Isometric diagram of disparate content in the TA 1-Capitol. Source: authors, 2025.

**Figure 12:**
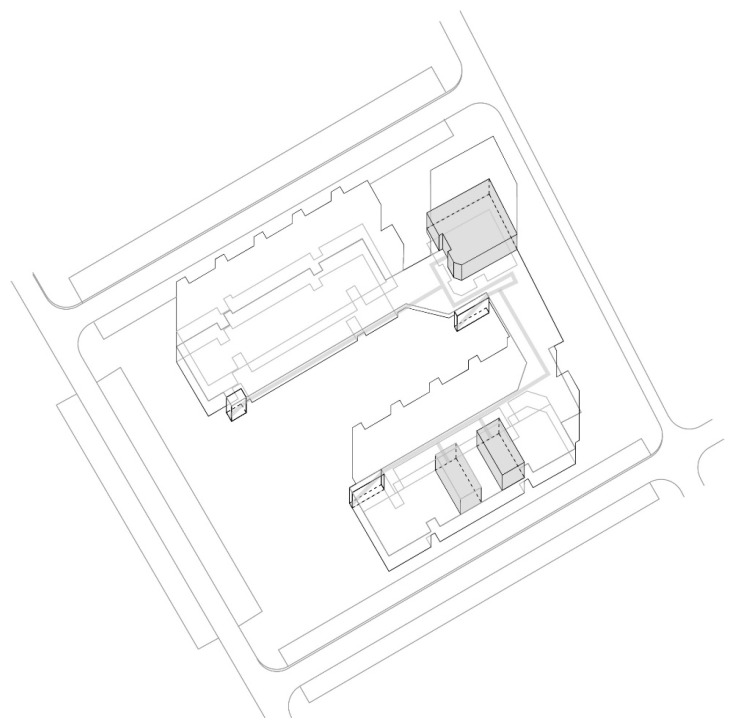
Isometric diagram of circulation in the TA 1-Capitol. Source: authors, 2025.

Trade area: Trade area 1;

Location: near Capitol Mall, Aerodrom municipality;

Year built: mid-1970s;

Healthcare facilities: Healthcare content that provides primary and secondary healthcare to the residents (Figure 10 and Figure 11) (Table 7);

Social interaction: The building’s circulation is organized so that the communications to the residential floors are separated from the rest of the visitors and patient movement (Figure 12).

The data have shown that transitioning healthcare into public and commercial facilities increases their contextual relevance and contributes to greater social inclusiveness, more accessible healthcare services, and sustainability.

The case studies elaborated upon through diagrams and tabular data demonstrate that outpatient clinics are uniquely positioned by integrating primary care, specialty services, and ancillary offerings in a single, easily accessible location. Most healthcare facilities have also expanded their operating hours to adapt to the users’ needs and to include working evenings and weekends. They are structured as private but often semi-private outpatient clinics, establishing the Public–Private Partnership (PPP) with the Health Insurance Fund (HIF). Their locations are strategically chosen, and they contribute to the medical practice’s financial sustainability.

The first isometric diagram analyzes the disparate levels of the shopping centers’ program content, and the second one analyzes patients’ circulation during their visits to the facility. Since different types of visitors use the building, it is crucial to anticipate pathway variations as qualitative variables reflecting visitors’ social characteristics inside the facility. These illustrations show the shopping centers’ flexibility and the possibilities to accommodate different content, such as medical services.

### 4.2. Analysis of Variance (ANOVA) Results

A statistical technique, Analysis of Variance (ANOVA), is used to compare means across multiple groups to determine whether there are significant differences between them.

This statistical technique can help determine whether architectural modification variations significantly impact public satisfaction, usability, and effectiveness in providing healthcare services. It is also integrated with other methodological frameworks used in this research.

Following an ANOVA test with F-values, the survey’s speculations are analyzed to give a probability (*p*-value) of whether or not the variations between the collective of healthcare users on given assumptions are statistically significant (Table 8).

Based on the positive difference and statistical significance of the arguments within the survey, it is concluded that the participants’ responses to the study are satisfactory regarding the following hypothesis:

- Time and transportation significantly affect the decision of the chosen medical institution;
- The proximity of walking distance significantly affects the decision of the chosen medical amenities;
- The presence of medical amenities in existing shopping centers gives significantly positive answers;
- The citizens in the residential area express their willingness to pay more for medical services rather than spend time waiting for “My Appointment” or travel to a public polyclinic.

According to their medical records, citizens express interest in specialized ambulances in their neighboring shopping centers. The results, which express significant differences between means for different locations and citizens’ satisfaction, suggest that the hypothesis offers substantial results in every area. Besides commercial programs in commercial buildings, healthcare also gives satisfactory results.

## 5. Discussion

Healthcare, according to Luhmann’s theory, is not a fully autonomous system but operates at the intersection of multiple functional systems. It primarily interacts with the medical system (science-based, healthy/sick logic), the economic system (profit/loss logic), the political system (government/opposition logic), and the legal system (legal/illegal logic). Healthcare depends on structural coupling, meaning it adapts to rules and constraints from these systems rather than functioning independently. This explains why healthcare reforms are complex; they must balance scientific, economic, political, and legal demands, which often have conflicting priorities.

Healthcare operates within a network of influences from multiple systems, while architecture provides the spatial conditions for effective healthcare delivery. Understanding these interactions through Luhmann’s framework allows for a more comprehensive approach to designing health-oriented, sustainable public spaces that support medical efficiency and societal well-being.

The location, accessibility, and design of health facilities play a key role in ensuring equitable access to health services. To ensure that communities receive appropriate care, facilities should be close to those most in need, well connected to transport networks, and financially accessible through affordable prices or universal insurance.

The design of buildings is also vital to ensuring that people feel welcome and respected, regardless of their socioeconomic status, race, ethnicity, language, values, gender, or cognitive abilities. By recognizing and meeting the community’s diverse needs, the architecture of public health buildings can promote a sense of dignity and inclusiveness.

Respecting and integrating community culture into building design helps create spaces that reflect people’s values and traditions, strengthening the connection between health services and the local population. This perspective is key in transforming public buildings into models that contribute to health and social sustainability [5].

The successful integration of public buildings into the urban fabric depends on their ability to address the needs of all citizens, regardless of their social or economic background. In the future, public buildings must function as catalysts for innovation, inclusiveness, and sustainability, providing environments that simultaneously promote health, ecology, and human values.

Public healthcare is part of a complex socioeconomic domain. The patient will inevitably have immediate access to medical treatment to suppress illness in its early stages. The redistribution of medical practice in facilities located in commercial shopping centers or residential buildings reduces the pressure on general and clinical hospitals. This transfer of patients to private medical institutions makes clinical trials more effective, especially in primary and secondary healthcare systems.

Shopping malls, as the subject of this study, accommodate about 4% of private practices in general healthcare and dentistry. Private outpatient clinics are also evident in residential buildings. Even 87% of medical facilities are in residential individual or collective buildings. The law and legislation treat outpatient clinics as commercial services in existing buildings [18]. According to national building legislation, approximately 30–40% of the gross area of the collective building can accommodate disparate programs such as commercial or medical services. The law and legislation are not precise in this area, so there are cases where X-ray diagnostics or extracting hazardous medical wastes appear in private outpatient clinics in residential collective buildings. In this case, the legislation is vague and does not recognize the risks of medical treatment procedures.

The presence of private polyclinics in shopping malls in Skopje illustrates structural coupling between the healthcare and economic systems, where medical services adapt to market-driven spatial constraints. However, as some of these facilities fail to meet the prescribed architectural standards (e.g., ventilation and lighting), they highlight a misalignment between medical regulations and commercial urban planning. From a Luhmannian perspective, this creates systemic inefficiencies, where the lack of cross-sectoral communication risks undermining public health standards and sustainable urban development.

## 6. Conclusions and Recommendations

This study emphasizes the benefits of medical amenities such as medical ordinations, polyclinics, or specialized hospitals in commercial shopping centers. Shopping centers are contemporary mixed-use buildings concentrated in the area of consumption. They are very well connected with the public transport system and usually have sufficient parking space. Shopping centers inherently have spatial primary and secondary hallways, flexible construction systems, and adequate vertical communications such as staircases, escalators, and elevators suitable for diverse visitors. These differences exemplify that shopping centers could serve the purpose of public healthcare better than residential buildings.

This phenomenon of hybridization between healthcare amenities and commercial stock exchange creates shopping centers as a transformative model toward sustainability. The presence of diverse users and programs emphasizes the social cohesion of the healthcare system and the economic sustainability of present shopping centers.

This research contributes to the broader discourse on resilient urban development by understanding the intersection between architecture, health, and social sustainability.

Integrating private polyclinics into shopping malls can enhance urban accessibility by reducing the need for separate medical trips and supporting mixed-use development. Private polyclinics in shopping malls offer convenient access, extended working hours, and modern medical services, making them ideal for quick check-ups and routine consultations. Their proximity to shops, pharmacies, and banks allows patients to combine medical visits with other daily tasks, enhancing practicality.

Some facilities fail to meet the prescribed architectural lighting, ventilation, and spatial organization standards, impacting patient well-being and safety. City planners should ensure sufficient public transport options and pedestrian-friendly infrastructure around these malls to minimize car dependency. Policies should encourage energy-efficient medical facilities and responsible waste management within commercial centers to promote sustainable healthcare access. Balancing commercial and essential services in urban planning can create more resilient, community-centered cities.

A multidisciplinary and participatory approach is essential, involving architects, urban planners, medical experts, and citizens in shaping healthier urban spaces. Finally, raising public awareness about sustainable architecture and its impact on health will be key to fostering long-term transformation.

## 7. Limitations of the Study

Crowded mall environments and limited space for specialized procedures make shopping malls less suitable for complex medical treatments. Additionally, higher patient turnover increases the risk of infections, especially during flu seasons. Patients may prefer larger medical centers with complete hospital infrastructure for serious health issues or surgical interventions.

A limitation of this study is the continuous transformation of the content within shopping centers, which is directly influenced by attendance and the overall relevance of the shopping center itself. The transformation and hybridization of public facilities to achieve social sustainability remain ongoing research topics.

## Figures and Tables

**Table 1:** The comprehensive social system (society as a whole) according to Luhmann. Source: Luhmann, N. *Social Systems*. Stanford University Press, 1995 [8].

The Comprehensive Social System (Society as a Whole)		
Functional Systems (Subsystems of Society)	Communication	Binary Code
**economy**	money	profit–oss
**politics**	power	government–opposition
**low**	legality	legal–illegal
**science**	truth	true–false
**media**	information	relevant–irrelevant
**education**	learning	qualified–unqualified
**religion**	faith	sacred–profane

**Table 2:** Timeline of healthcare system decentralization.

Timeline of Healthcare System Development	
21st century in Europe and the US	Decentralization, networks of mostly smaller healthcare facilities.
Second half of 20th century in Yugoslavia	Centralized health system.
1991–2007 in Macedonia	Highly centralized health system.
2007 ongoing reforms in Macedonia	Decentralization—establishing a Public–Private Partnership (PPP) among healthcare providers.

**Table 3:** Distribution of private practices in general healthcare and dentistry. Source: https://www.mojtermin.mk/map/ustanovi (accessed on 24 December 2024).

	Private Health Practices	Private Health Premises in Public Health Centers	Private Health Facilities in Shopping Centers	Private Healthcare Premises Integrated into Other Facilities
Center municipality	40%	3.5%	12%	95%
Aerodrom municipality	38%	10%	3.75%	86%
Karposh municipality	22%	16%	6.8%	77%
In total	100%	9%	4%	87%

**Table 4:** Qualitative comparative data report on the medical staff questionnaire.

**Decision Factor** **According to the Questionnaire**	**Medical Practices in SC** **Shopping Center (SC)**							
	**Olimpiko**				**Kapistec**	**TA 1-Capitol**		
	**Dentistry**	**Dentistry**	**Dentistry**	**Eye** **Clinic**	**Special Hospital**	**General Practice**	**Dentistry**	**Polyclinic**
Favorable price of renting the space	●	●				●	●	
In ownership			●	●	●			●
High frequency	●	●	●	●	●	●	●	●
Space area					●			●
Proximity to the neighborhoods	●	●	●	●		●	●	
Only work with appointments	●	●	●	●	●	●	●	
Available healthcare services at any time								●

●: Dots represent the presence or absence of Med.Practice according to questions.

**Table 5:** Healthcare facilities analysis in SC Olimpiko: service offerings.

Service Offering SC Olimpiko	1. General Dentistry Outpatient Clinic–Radarani, Skopje	2. Еye Outpatient Clinic and Optics– Zenit, Skopje	3. General Dentistry Outpatient Clinic–Estetic, Skopje	4. General Dentistry Outpatient Clinic–32, Skopje
Area–m^2^	25	78	50	25
Monday–Friday hours	8:00 a.m.–4:00 p.m.	8:00 a.m.–4:00 p.m.	8:00 a.m.–7:30 p.m.	9:00 a.m.–1:00 p.m. 4:00 p.m.–7:00 p.m.
Saturday–Sunday hours	closed	closed	9:00 a.m.–2:00 p.m.	9:00 a.m.–1:00 p.m.
Specialties available	general dentistry	Specialist Ophthalmology Clinic	general dentistry	general dentistry and prosthetics
Provider staffing	- dentist and medical nurse	- ophthalmologist and optician - technicians.	- dentist and medical nurse	- dentist, prosthodontist, and medical nurse
Private/public/semi-private in cooperation with the HIF	private	Semi-private	private	private

**Table 6:** Healthcare facilities analysis in SC Kapishtec: service offerings.

Service Offering SC Kapishtec	1. Special Hospital for Eye Diseases– d-r Serafimovski, Skopje
Area–m^2^	1286
Monday–Friday hours	8:00 a.m.–4:00 p.m.
Saturday–Sunday hours	closed
Specialties available	Specialist Ophthalmology Clinic
Provider staffing	- ophthalmologists and medical nurses; - technicians.
Private/public/semi-private in cooperation with the HIF	private

**Table 7:** Healthcare facilities analysis in TA 1-Capitol: service offerings.

Service Offering TA 1-Capitol	1. Polyclinic Tospash, Skopje	2. General practice Helio Medika, Skopje	3. General Dentistry Outpatient Clinic–d-r Babamova, Skopje
Area–m^2^	260	89	83
Monday–Friday hours	(Mon.; Tue.; Thur.; Fri.) 8:00 a.m.–3:00 p.m. (Wednesday) 8:00 a.m.–5:30 p.m.	7:30 a.m.–7:00 p.m.	8:00 a.m.–8:00 p.m.
Saturday–Sunday hours	closed	closed	8:00 a.m.–2:00 p.m.
Specialties available	- specialist ophthalmology and otorhinolaryngology practice; - biochemistry laboratory; - general medicine	- general medicine	- general dentistry
Provider staffing	- ophthalmologist, otolaryngologist, physicians, medical nurses; - technicians.	- physicians and medical nurses	- dentist and medical nurse
Private/public/semi-private in cooperation with the HIF	Semi-private	Semi-private	Semi-private

**Table 8:** One-way ANOVA regarding different social groups. Behavior of participants.

	Sum of Squares	Df	Mean Square	*f* -Ratio Value	*p* -Value Significance
The impact of the distance on the time required for transportation to the healthcare facility	46.4182 ***	4	11.6405	6.841	0.00003
The importance of the proximity of healthcare programs within walking distance	39.1269 ***	1	39.1269	33.2844	0.000
The necessity for the presence of healthcare services in existing shopping centers near the place of residence	16.398 *	3	5.466	3.2697	0.0218
The importance of the proximity of public healthcare facilities within shopping centers	54.5579 ***	1	54.5579	52.5229	0.000
The importance of the proximity of private healthcare facilities within shopping centers	0.535	1	0.535	0.3711	0.5435
The importance of the proximity of the semi-public healthcare facilities within shopping centers	34.4915 ***	1	34.4915	27.8611	0.000
The necessity of specific healthcare services (dentistry, biochemical laboratory, ophthalmology, otorhinolaryngology, gynecology, general practitioner) in the nearest shopping center	50.7264 *	6	8.4554	2.3736	0.0287

Notes: * Difference is statistically significant (*p* < 0.05); *** difference is statistically significant (*p* < 0.001).

## Data Availability

The original contributions presented in this study are included in the article. Further inquiries can be directed to the corresponding author.
